# Multidisciplinary Guidance to Care for Persons With Xylazine-Associated Wounds

**DOI:** 10.1093/ofid/ofaf299

**Published:** 2025-05-15

**Authors:** Wei-Teng Yang, Jessica A Meisner, Christina Maguire, Kelly E Dyer, Rachel McFadden, Ashish P Thakrar, Drew T Dickinson, Deanna Berg, Ave Preston, Michael Z David, Jeanmarie Perrone, Naasha Talati, Kathleen O Degnan

**Affiliations:** Division of Infectious Diseases, Department of Medicine, University of Pennsylvania Perelman School of Medicine, Philadelphia, Pennsylvania, USA; Center for Addiction Medicine and Policy, University of Pennsylvania, Philadelphia, Pennsylvania, USA; Division of Infectious Diseases, Department of Medicine, University of Pennsylvania Perelman School of Medicine, Philadelphia, Pennsylvania, USA; Prevention Point Philadelphia, Philadelphia, Pennsylvania, USA; Department of Pharmacy, Penn Presbyterian Medical Center, Philadelphia, Pennsylvania, USA; Division of Infectious Diseases, Department of Medicine, University of Pennsylvania Perelman School of Medicine, Philadelphia, Pennsylvania, USA; Prevention Point Philadelphia, Philadelphia, Pennsylvania, USA; Department of Emergency Medicine, University of Pennsylvania Perelman School of Medicine, Philadelphia, Pennsylvania, USA; Center for Addiction Medicine and Policy, University of Pennsylvania, Philadelphia, Pennsylvania, USA; Division of General Internal Medicine, Department of Medicine, University of Pennsylvania Perelman School of Medicine, Philadelphia, Pennsylvania, USA; Department of Pharmacy Services, Hospital of the University of Pennsylvania, Philadelphia, Pennsylvania, USA; Department of Pharmacy, Penn Presbyterian Medical Center, Philadelphia, Pennsylvania, USA; Department of Wound and Ostomy Nursing, Hospital of the University of Pennsylvania, Philadelphia, Pennsylvania, USA; Division of Infectious Diseases, Department of Medicine, University of Pennsylvania Perelman School of Medicine, Philadelphia, Pennsylvania, USA; Center for Addiction Medicine and Policy, University of Pennsylvania, Philadelphia, Pennsylvania, USA; Department of Emergency Medicine, University of Pennsylvania Perelman School of Medicine, Philadelphia, Pennsylvania, USA; Division of Infectious Diseases, Department of Medicine, University of Pennsylvania Perelman School of Medicine, Philadelphia, Pennsylvania, USA; Division of Infectious Diseases, Department of Medicine, University of Pennsylvania Perelman School of Medicine, Philadelphia, Pennsylvania, USA

**Keywords:** illicitly manufactured fentanyl, multidisciplinary care, opioid use disorder, persons who use drugs, xylazine-associated wounds

## Abstract

Xylazine, an adulterant of unregulated opioid supplies, is increasingly prevalent in the United States and associated with distinctive wounds. Xylazine-associated wounds (XAWs) are primarily noted in the extremities and are not always associated with injection drug use. XAWs are often chronic and can become superinfected, posing a great challenge to clinical care. We share multidisciplinary guidance to care for persons with XAWs: (1) substance use disorder treatment and longitudinal multidisciplinary care including addiction medicine, wound care, infectious diseases, and surgery are imperative; (2) avoid aggressive debridement; (3) administer empirical antibiotics for methicillin-resistant *Staphylococcus aureus* (MRSA)– and group A *Streptococcus* (GAS)–infected wounds, specifically oral trimethoprim-sulfamethoxazole for MRSA and oral β-lactams for GAS; (4) administer intravenous daptomycin to reduce the discomfort and challenges associated with frequent phlebotomy for vancomycin therapeutic drug monitoring; and (5) create explicit contingency antibiotic plans with potential use of linezolid, tedizolid, or dalbavancin for patient-directed hospital discharge.

## Teaser

Xylazine-associated wounds may be increasingly encountered as xylazine becomes more prevalent in illicit substances. This review summarizes the current understanding of and multidisciplinary treatment approaches to these wounds. Studies are urgently needed to inform best practices.

Xylazine, an alpha-2 adrenergic agonist and veterinary tranquilizer, is increasingly prevalent as an adulterant of illicitly manufactured fentanyl (IMF) in the United States (US). The prevalence is highest in the northeastern US, but it has been found in drug seizures in 48 states as of 2023 [1–5]. It is associated with distinctive wounds [6–9]. Philadelphia, where xylazine was first detected in the drug supply in the US [10], remains the epicenter of xylazine-associated morbidity and mortality [11 ]. In 2023, xylazine was found in 99% of IMF samples in Philadelphia. The weight percentage of IMF in Philadelphia drug samples remained stable (15%), but the weight percentage of xylazine has increased (34% in 2022 to 46% in 2023) [12]. Xylazine-associated wounds (XAWs) are often large and chronic and can become superinfected. Since 2020, wound care clinics and hospitals in Philadelphia have observed a dramatic increase in utilization of wound care services [7], emergency department (ED) visits, and hospitalizations related to XAWs [13]. For the diagnosis of drug-associated skin and soft tissue infections (SSTIs), the number of hospitalizations in Philadelphia hospitals increased from 1791 in 2020 to 2881 in 2022, and the number of ED visits increased from about 125 per quarter in 2020 to about 300 per quarter in 2022 [13]. XAWs may be the latest wave of drug use–associated skin lesions affecting the health of persons who use drugs (PWUD) and healthcare costs [14–17 ]. Here we summarize our institutional clinical observations and strategies to care for XAWs.

## METHODS

The clinical observations and management recommendations are based on institutional practices and consensus in Philadelphia [6, 7, 18–24]. These recommendations were approved by institutional experts from wound care, addiction medicine, infectious diseases (ID), and surgery.

## CURRENT UNDERSTANDING OF XAWs

**Figure 1.**
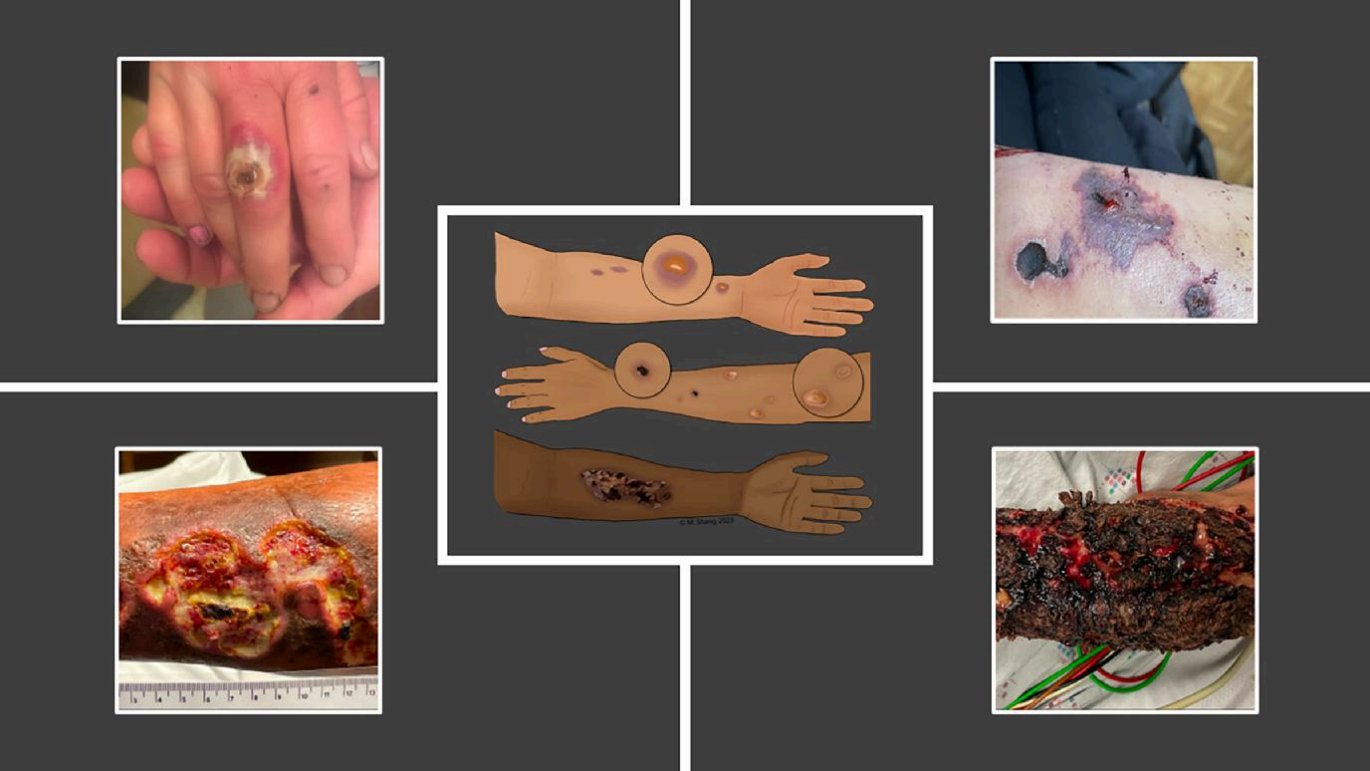
Xylazine-associated wounds (XAWs). Center: a schema of different stages of XAWs. Courtesy of Dr Margaret Shang [25, 28]. Upper left: a coin-sized painful blister with a central eschar. Upper right: painful dark necrotic areas with intact skin, resembling unstageable deep tissue pressure injury. Lower left: angulated open wounds with small areas of eschars and islands of healthy tissue. Lower right: a wound with extensive eschar. Wound pictures from Lutz et al [6] and McFadden et al [7] with permission to reuse.

The pathogenesis of XAWs remains unclear. Current observations suggest that XAWs do not appear to be infected in their early stage. This is different from the skin lesions related to heroin use that typically start as cellulitis or abscesses [7, 13]. We observe 2 types of XAWs. First, XAWs at or close to injection sites start as small blisters or bruises, which may coalesce and harden into dry eschar and eventually ulcerate to form large wounds [7, 23, 25, 26]. The ulcers are often heterogenous and contain full-thickness necrosis interspersed with islands of viable tissue [7, 13, 23, 26, 27] (Figure 1). These wounds share similarities with skin lesions from burn or pressure injuries resulting from tissue necrosis [13]. Underlying tendons or bones may be exposed and infected. In severe cases, the destruction and infection resulting from XAWs lead to limb loss [29–32]. Another type of XAWs occurs distant from injection sites and may develop when people only snort or smoke substances [7, 13]. These XAWs often present as coin-sized ulcers covered with friable eschars [7]. Additionally, they are observed at sites of minor injuries (eg, skin trauma from falls or insect bites) [13, 33]. They may also ulcerate to form larger wounds. Given the heterogeneity of wound types and ongoing stigma related to wounds [34], it is important to proactively ask PWUD about XAWs. In a case series, most XAWs had predominantly devitalized tissue and were located on extremities, especially on extensor surfaces. Forty-nine percent of XAWs were present for >1 month, and they tended to be larger [6]. Atypical locations of XAWs such as sternum [35] and scalp [36 ] have also been reported.

XAWs are currently diagnosed clinically. In Philadelphia, as nearly all IMF samples contain xylazine, the diagnosis is assumed for PWUD with characteristic wounds without alternative explanations. It is important to consider comprehensive differential diagnoses first, including wounds from injuries or typical SSTIs. In other settings where xylazine–IMF coexistence is less prevalent, we recommend sending urine xylazine test to aid the diagnosis of XAWs. The terminal half-life of xylazine in urine is approximately 12 hours, suggesting a window of detection of approximately 2 days when using highly sensitive mass spectrometry [37]. Commercially available xylazine screening test strips, such as those developed by BTNX Inc. (Pickering, Ontario, Canada), may help rapid detection of xylazine in urine or drug samples where confirmatory laboratory testing for xylazine [38, 39] is not available. However, their sensitivity is diminished when xylazine is not the primary substance detected [40], and lidocaine may interfere with the assay, leading to false positivity [41].

## MULTIDISCIPLINARY APPROACH TO CARE FOR PERSONS WITH XAWs

**Figure 2.**
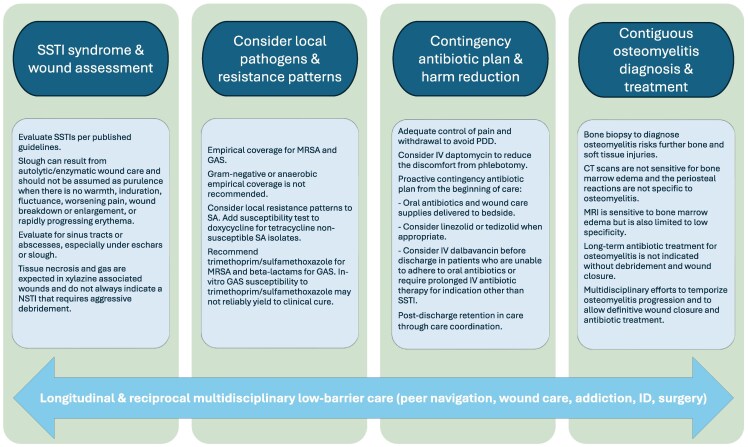
A multidisciplinary framework to care for persons with xylazine-associated wounds. Abbreviations: CT, computed tomography; GAS, group A *Streptococcus*; ID, infectious diseases; IV, intravenous; MRI, magnetic resonance imaging; MRSA, methicillin-resistant *Staphylococcus aureus*; NSTI, necrotizing soft tissue infection; PDD, patient-directed discharge; SA, *Staphylococcus aureus*; SSTI, skin and soft tissue infection.

Our multidisciplinary care team includes members from peer navigation and care coordination, wound care, addiction medicine, ID, and surgical specialties (plastic surgery, orthopedic surgery, and podiatry). Because of clinical similarities including full-thickness tissue necrosis with superinfection, tissue exposure, and environmental contamination of the wounds, a multidisciplinary approach to XAWs (Figure 2) was adapted from a published approach to contiguous pelvic osteomyelitis in sacral ulcers [42].

### Peer Navigation

Peer navigators, or certified recovery specialists, are integral to care for PWUD [43]. They have lived experiences with opioid use disorder (OUD) and/or infectious complications, as well as knowledge of the local drug scene and community resources. In healthcare settings, their lived experiences coupled with the removal from medical culture and hierarchy often facilitate bonding between the treatment teams and patients. Outside of healthcare settings, they continue to serve as peer mentors. They deliver essentials (food, clothing, phone, and vouchers) and facilitate linkage to social services, housing, and appointments. While many peers only work in inpatient or outpatient settings, a longitudinal peer–patient relationship that starts in healthcare settings and continues into the communities may be helpful to reduce gaps in care [44].

### Wound Care

Two frameworks to care for chronic wounds are adapted to care for PWUD with chronic XAWs [45, 46] (Table 1). The SPECIAL framework [45] was initially proposed for palliative care patients who may not tolerate aggressive curative interventions for chronic wounds. However, its principles (S: stabilizing wounds; P: preventing new wounds; E: eliminating odor; C: control pain; I: infection prevention; A: absorbent dressing; L: lessen dressing changes) align with patient-centered and harm reduction goals. We adopt it to promote the comfort of patients. The DIME framework summarizes the principles of wound bed preparation for the treatment of chronic wounds (D: devitalized tissue; I: infection/inflammation; M: moisture: E: edge preparation) [46]. This framework serves as a technical guide. In brief, wound care should include cleaning with nonirritant solution (water or soap, avoiding peroxide or alcohol), maintaining wound moisture with nonadherent primary dressing to the wound bed, absorbing drainage with secondary dressing, and securing the entire dressing without causing compression [7] (Table 2). We use the same wound care principles for infected XAWs.

**Table 1.** SPECIAL and DIME Frameworks to Care for People With Xylazine-Associated Wounds

Framework	Description
SPECIAL framework to promote comfort in people with chronic wounds [7, 18, 45]	
Components of SPECIAL	Treatment strategies
** S**tabilize wounds; **s**tigma reduction [47]	Regular wound care; nutrition; housing and hygiene; limit sharp debridement; use nonjudgmental patient-first language and create a safe space.
** P**reventing new wounds; **p**rivacy	Safe injection; alternative route of drug consumption; avoid/limit injection in/around wounds; limit sharp debridement; ask permission and respect privacy before offering services.
** E**liminating odor; **e**mpathy	Maintain composure; offer aromatherapy inhalers or change trash frequently [7]; inquire about prior successes and failures to identify appropriate individualized wound care.
** C**ontrol pain	Give adequate analgesics before dressing change when possible; soak old dressing before changing; allow patient-directed dressing change; BTM placement; methadone split dosing if possible [48].
** I**nfection prevention	Education of infectious symptoms and signs; topical or oral antibiotics when appropriate; dispense in lanyard-attached plastic pockets to avoid loss or theft [7].
** A**bsorbent dressing	Offer ample secondary dressing supply to reduce drainage.
** L**essen dressing changes	Reduce dressing change frequency to reduce pain and avoid trauma to wound edge; balance this with wound healing.

DIME framework as a technical framework to care for chronic wounds [46]		
Components of DIME	Wound characteristics	Treatment strategies
** D**evitalized tissue	Necrotic, hypoxic wound bed with eschar (dry, dark, firm, and adherent) or slough (wet, yellow/white; soft; stringy) [18].	Autolytic debridement via dressing product and immune system: honey; hydrogel Enzymatic debridement: collagenase (Santyl) Sharp debridement: scissors; curettes; blades or scalpels Surgical debridement: done in OR
** I**nfection/**i**nflammation	Contaminated/colonized wounds (eg, biofilms) with immune responses leading to chronic inflammation, or local infection with foul odor, peri-wound erythema, tenderness, warmth, edema, and discharge.	Silver sulfadiazine (Silvadene) and PHMB are the preferred topical antimicrobial because they are affordable and provide moisture. Use published guidelines [49] to diagnose wound infection and use systematic antibiotics judiciously.
** M**oisture balance	Both drainage/maceration and desiccation lead to poor wound healing.	Add moisture to dry wounds (hydrogel; Silvadene ointment); absorb drainage from wet wounds (calcium alginate or hydrofiber dressings; abdominal pads; nonadherent pads). Barrier ointments applied to intact peri-wound tissue (eg, zinc, petroleum).
** E**dge of the wound	Hypergranulation tissue, wound edge maceration or sinus tracts would impede wound healing.	Remove hypergranulation tissue; sinus track debridement; nonstick dressing and barrier cream/ointment to protect wounds; absorbent dressing to avoid wound edge maceration; avoid compression when securing dressing.

The first letter of each component in SPECIAL and DIME frameworks was bolded to highlight the acronyms. Abbreviations: BTM, biodegradable temporizing matrix; OR, operating room; PHMB, polyhexamethylene biguanide.

**Table 2.** Basic Wound Dressing

Wound Cleansing	Primary Dressing (Maintains Moisture; Facilitates Debridement)	Secondary Dressing (Absorbs Drainage)	Reenforcing Dressing
Wash with clean water, saline, mild soap, or hypochlorous acid soaks Soak old dressing before removal Avoid peroxide and alcohol Gently wipe with wet gauze	Choose 1 barrier ointment to apply to gauze with ointment facing the wound bed: Petroleum Silver sulfadiazine Choose 1 of the following dressings: Impregnated gauze such as Adaptic (oil emulsion) or Xeroform (bismuth tribromophenate and petroleum) for dry to regular wounds Hydrofiber or calcium alginate with or without silver for wet wounds Use autolytic (honey or hydrogel) or enzymatic (collagenase) products in the presence of devitalized tissue	Abdominal pad or extra dry gauze Change secondary dressing without removing primary dressing if there is copious drainage	Choose 1: gauze roll; ACE bandage; athletic wrap; socks; shoes; gloves Secure dressing but avoid compression

Please refer to “Recommendations for caring for individuals with xylazine-associated wounds” by the Philadelphia Department of Public Health for commonly used and affordable wound supplies [18].

Autolytic or enzymatic wound care products should be applied to the primary dressing to facilitate debridement of devitalized tissue (eschar or slough) (Table 1). Notably, both products increase wound drainage (slough). Without signs of local infection, the drainage should not be assumed to be purulent. It only requires removal, and antibiotic therapy is usually not necessary. Sharp debridement should be limited to avoid the loss of healthy tissue in XAWs [19]. It is observed that the islands of healthy tissue within XAWs possess great healing potential; therefore, aggressive, sharp debridement may undermine tissue regeneration and result in larger wounds [18, 23]. A synthetic biodegradable dermal substitute, biodegradable temporizing matrix (BTM; Novosorb BTM, PolyNovo, Melbourne, Australia), has been recommended to cover large XAWs and promote healing [18, 19, 23, 50]. In most chronic wounds, especially those containing devitalized tissue, the presence of biofilm should be assumed. Wound biofilms comprise noxious aggregates of microorganisms that are protected from host immune defenses and even systemic antibiotics and impair healing through hyperinflammation [51]. Biofilm disruption requires the application of topical antimicrobial agents (eg, silver, polyhexamethylene biguanide) directly to the wound bed, in addition to debridement.

Many PWUD with XAWs do not immediately cease substance use for multiple reasons: for example, fear of pain or withdrawal; inadequate access to addiction treatment; or substance use as a coping strategy. Instead of punitive approaches, such as withholding necessary care in the setting of ongoing substance use, harm reduction principles should be employed [7, 18, 33]. Ongoing substance use is not an absolute contraindication for interventions including BTM. Injection in or around XAWs is common, possibly due to a lack of venous access, local analgesic effect, or fear of developing new XAWs [13, 26]. It may impair healing and lead to early-stage XAW (eg, new blisters or eschars) around older XAWs. Because XAWs can occur distant from injection sites and even in the absence of injection of substances (eg, with insufflation or smoking), harm reduction discussions must acknowledge the risk balance between new XAWs that may still occur after refraining from peri-wound injection and the worsening of existing XAWs.

Patients' experiences with various wound care products should be considered and their wound care preferences respected. Providing patients with wound supplies (Table 2) during hospitalization is important in the event of patient-directed discharge (PDD). Wound supplies should be affordable, and information on community wound care services should be given to PWUD. To address the fear of limb loss resulting in delayed care seeking [34], it is important to share with patients examples of successful wound healing [35, 50, 52]. A recent case series [50] reported 4 patients with a high proportion of housing insecurity, ongoing injection, and exposure of deep tissues who received wound care with imperfect follow-up. Their XAW improved significantly after 90–320 days. These examples can motivate people to continue wound and OUD care.

### Addiction Medicine

People with XAWs should be treated adequately for pain and withdrawal to facilitate the evaluation of XAWs and the retention in care [53]. As an adjuvant to methadone or buprenorphine treatment for withdrawal, short-acting opioid agonists for pain and withdrawal are feasible, can be implemented safely [53–55], and might reduce the rate of PDD [54]. In a case series of patients using IMF in Philadelphia, the median daily dose of short-acting opioid agonists in addition to methadone or buprenorphine ranged between 200 and 320 oral morphine milligram equivalents in the first 3 days of hospitalization. All but 1 patient who remained hospitalized were discharged with a medication for opioid use disorder (MOUD) [54].

Experts have raised concerns for a potential xylazine withdrawal syndrome characterized by elevated heart rate or blood pressure and anxiety, due to xylazine's central α-2 adrenergic agonist effects. Nevertheless, an objective and distinct withdrawal syndrome has not been identified. In a study of hospitalized patients who used IMF and had urine toxicology tests within 48 hours, those with xylazine detected in urine compared to those without did not have significantly different vital signs or a distinct withdrawal syndrome. The evaluation of anxiety as a withdrawal symptom from xylazine in this study was confounded by high rates of withdrawal from fentanyl, benzodiazepines, and alcohol [56].

### Infectious Diseases Evaluation

Infectious diseases evaluation for PWUD with XAWs follows the conceptual framework shown in Figure 2. A full spectrum of SSTIs and wound conditions needs to be considered. Typical SSTIs including cellulitis, abscesses, and necrotizing soft tissue infections (NSTIs) should be treated according to published guidelines [57]. According to guidelines, purulence or slough along with warmth, induration, fluctuance, worsening pain, wound breakdown or enlargement, or rapidly progressing erythema should be used to diagnose infection of a chronic wound [49] and to avoid overuse of systemic antibiotics (Table 3, Supplementary Table 1). As mentioned previously, drainage is expected with autolytic/enzymatic wound care and should not be automatically assumed to be purulent. Notably, tissue necrosis is a characteristic of XAWs, and the tissue defects in XAWs may contain gas, which may appear on radiographic studies. It is imperative to differentiate tissue necrosis associated with XAWs from necrosis resulting from an acute NSTI to avoid overdiagnosis of acute NSTI and unnecessary surgical debridement [19].

**Table 3.** Antibiotic Guidance for Skin and Soft Tissue Infections Related to Injection Drug Use

Step 1: Determine an SSTI syndrome and consider local pathogen burden and antimicrobial resistance.
Purulent cellulitis/abscess → IV vancomycin or daptomycin; oral TMP-SMX [57]
Nonpurulent cellulitis → IV cefazolin; oral amoxicillin 1 g q12h; cefadroxil 1 g q12h; cephalexin 500 mg q6h [57]
Infected wounds, including XAW → proceed to the following steps:

Step 2: Assess the condition of XAW.		
Healthy wound	Wound with eschar	Infected wound
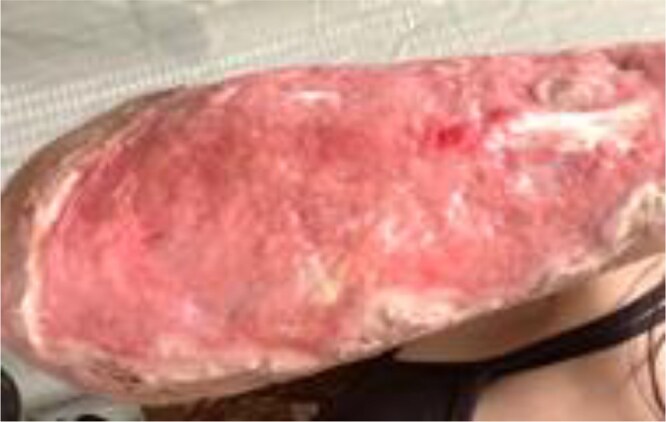	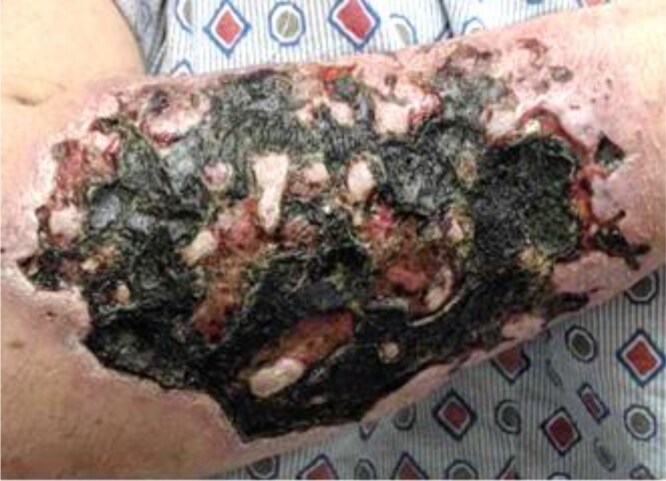	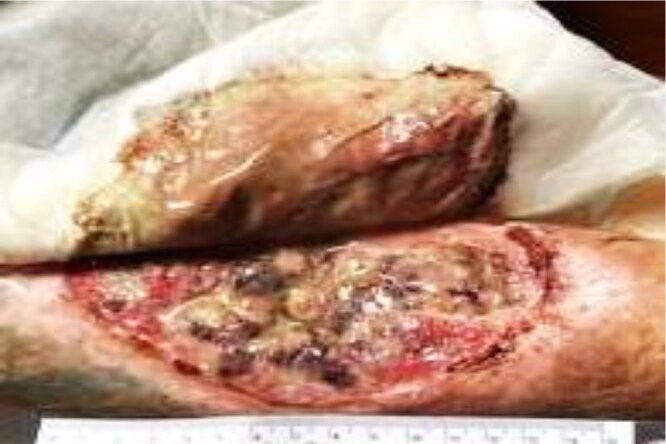
Antibiotics not needed. Wound care.	If there is no purulence, warmth, induration, fluctuance, worsening pain, wound breakdown or enlargement, or rapidly progressing erythema [49]: Antibiotic not indicated Intensive wound care Consider sharp debridement of eschar if there is significant burden not responding to autolytic/enzymatic debridement	Purulence or slough along with warmth, induration, fluctuance, worsening pain, wound breakdown or enlargement, or rapidly progressing erythema [49]. Refer to the main text that slough can result from autolytic/enzymatic wound care that is independent from infection: Antibiotic indicated Rule out deep structure infection

Step 3: Identify a clinical syndrome associated with XAW.			
Superimposed NSTI in XAW: Avoid overdiagnosis (refer to main text ID section) Surgical debridement Empirical antibiotics per published guidelines [57] and include MRSA and GAS coverage	Cellulitis/abscess in XAW without exposure of tendons or bones: Limited surgical debridement only for abscesses/sinus tracts/superinfected necrotic tissue Treat as common cellulitis/abscess (5–7 d) Rule out deep source and review culture results with lack of improvement Intensive wound care	Cellulitis/abscess in XAW with exposure of tendons or bones or osteomyelitis: Limited surgical debridement only for abscesses, sinus tracts, or superinfected necrotic tissue Treat as common cellulitis/abscess (5–7 d) Rule out deep source and review culture results with lack of improvement Prolonged antibiotic therapy is futile until the XAW can be debrided and covered Intensive wound care	Cellulitis/abscess in XAW with bacteremia or endocarditis: Stronger consideration for surgical debridement if abscesses, sinus tracts, or superinfected necrotic tissues are thought to be the source of bacteremia Duration of antibiotic therapy per bacteremia/endocarditis treatment Intensive wound care

Step 4: Wound culture to guide definitive antibiotics.
Empiric antibiotic is appropriate if there is no tissue sample to be cultured.
Limit debridement and sample only from NSTI, abscess, infected necrotic tissue, or deep tissue cultures (skin, soft tissue, bone) in the setting of definitive debridement and wound coverage.
Avoid superficial wound swabs for culture. Take culture from OR bedside I&D to avoid superficial bacterial colonization.

Step 5: Empiric antibiotics for MRSA and GAS before culture results are available (choose 1 row). Adjust treatment per culture results.	
**IV** vancomycin with trough 10–15 μg/mL	
**IV** daptomycin 6 mg/kg/d if any of the following: (1) difficult venous access; (2) refusing creatinine or vancomycin level blood draws; (3) requiring vancomycin dosing more frequent than q8h; (4) subtherapeutic vancomycin level despite high-dose q8h dosing; or (5) impending discharge to outpatient parenteral antibiotic therapy with current IV vancomycin q8h	
**Oral** TMP-SMX 8 mg/kg/d divided into 2–3 daily doses **AND 1 β-lactam drug from the right column**	Amoxicillin 1 g q12h
	Cefadroxil 1 g q12h
	Cephalexin 500 mg q6h
**Oral** linezolid 600 mg q12h or tedizolid 200 mg q24h	
**IV** dalbavancin 1500 mg once, followed by 1000 mg weekly for the remaining severe infection treatment duration. For SSTI, 1 dose is adequate. Subsequent doses are only needed for those with bacteremia, infective endocarditis, osteomyelitis, or discitis/epidural abscess.	

Photographs of the wounds courtesy of the Penn Center for Addiction Medicine and Policy [20].

Abbreviations: GAS, group A *Streptococcus*; I&D, incision and drainage; ID, infectious diseases; IV, intravenous; MRSA, methicillin-resistant *Staphylococcus aureus*; NSTI, necrotizing soft tissue infection; OR, operating room; q6h, every 6 hours; q8h, every 8 hours; q12h, every 12 hours; q24h, every 24 hours; SSTI, skin and soft tissue infection; TMP-SMX, trimethoprim-sulfamethoxazole; XAW, xylazine-associated wound.

Local pathogens and antimicrobial resistance among PWUD need to be considered to inform the selection of empirical antibiotic regimens. We only recommend wound culture from deep samples (operating room or bedside debridement) to avoid detection of only superficial bacterial colonization. Following the principle of limited debridement, samples should be from NSTI, abscess, infected necrotic tissue, or deep tissue cultures (soft tissue, bone) in the setting of definitive debridement and wound coverage. Wound cultures from 81 PWUD with XAWs in 1 local academic hospital showed 56% with growth of methicillin-resistant *Staphylococcus aureus* (MRSA), 37% with growth of group A *Streptococcus* (GAS), and 10% with growth of methicillin-susceptible *S aureus*. Gram-negative organisms grew in 12% of the wounds, the majority of which involved joints. Anaerobic organisms grew in 14% of the wounds [22]. Given these results, our institutional practice includes empiric coverage of MRSA and GAS for infected XAWs. We do not empirically cover gram-negative or anaerobic organisms for SSTIs. Another study [21] of the *Staphylococcus aureus* (SA) resistance patterns from people who used IMF who presented with an SSTI to local academic hospitals showed 40% clindamycin resistance and 37% tetracycline resistance. Resistance to trimethoprim-sulfamethoxazole (TMP-SMX) was 6% among SA isolates. Therefore, we recommend TMP-SMX as the preferred empirical oral agent for SA. Additionally, microbiological data showed that 70% of tetracycline-nonsusceptible SA isolates retained susceptibility to doxycycline [58]. We recommend additional susceptibility testing to doxycycline for tetracycline-nonsusceptible SA isolates. For GAS infections, we recommend using β-lactam agents. Despite in vitro susceptibility of GAS to TMP-SMX [59, 60], TMP-SMX for GAS infection may not result in reliable clinical cure [61], especially considering recent outbreaks of invasive GAS infections in PWUD [62–64] (Table 3).

Harm reduction and shared decision-making should be incorporated into antibiotic treatment plans. To reduce the discomfort from challenging or frequent phlebotomy for intravenous vancomycin therapeutic drug monitoring [65], we recommend using daptomycin in lieu of vancomycin in selected clinical scenarios (Table 3). PWUD often leave hospitals via PDD [66, 67], and we advocate for continuation of antibiotic treatment. With care coordination, published experiences have suggested that many patients after PDD continue to take antibiotics and MOUD [68–70]. Strategies to facilitate retention in care include explicit contingency antibiotic plans from ID [69]; antibiotics delivered bedside; offering linezolid/tedizolid or dalbavancin when appropriate; and updating locating information. One concern with use of linezolid in PWUD is serotonin syndrome. Many opioids have serotonergic effects [71, 72], and the serotonergic effect of IMF is unknown. Many PWUD also take multiple serotonergic medications [73]. One retrospective study reported only 2 possible cases of serotonin syndrome among 492 patients who received concurrent linezolid and methadone/buprenorphine from 2015 to 2019 [74]. In both cases, patients took methadone for >3 days and had >3 other concurrent serotonergic medications. Given their excellent activity against gram-positive organisms and oral formulation, linezolid or tedizolid should be considered to treat infections in PWUD. However, insurance prior authorization may be a barrier to their use. Dalbavancin has been used off-label for gram-positive infections in PWUD [75–77] and in a recent trial showed comparable efficacy to standard of care in complicated SA bacteremia [78]. A common strategy is to infuse a dose of dalbavancin upon PDD, with subsequent doses arranged in outpatient infusion centers if indicated. However, many health systems restrict dalbavancin for outpatient use only, which is a barrier. Furthermore, postdischarge administrative requirements and costs for subsequent doses can be substantial [79].

Except for severe cases [30–32], diagnosis and management of contiguous osteomyelitis from XAWs are challenging. Many cases involve limb osteomyelitis. As with contiguous pelvic osteomyelitis from sacral ulcers, bone histopathological analysis is the gold standard for diagnosis. However, bone biopsy risks further damaging the bones and soft tissues in XAWs. Computed tomographic (CT) scans and X-rays are insensitive to bone marrow edema (correlates with osteomyelitis), and etiologies of periosteal reactions may be nonspecific including bone remodeling and inflammation from surrounding soft tissue infection. On the other hand, magnetic resonance imaging may be oversensitive and nonspecific to diagnose osteomyelitis [42]. Like contiguous pelvic osteomyelitis from sacral ulcers, long-term antibiotic treatment is futile without debridement and wound closure [42]. Periodic local infection “flares” should be treated as SSTIs arising from chronic wounds with short-term (5–7 days) antibiotics. Consequently, the management of chronic XAWs with exposed tendons or bones needs to focus on intensive multidisciplinary care coordination and preservation of tissue and limb function until eventual wound closure and definitive antibiotic therapy [19].

### Surgery

A comprehensive discussion of surgical techniques is beyond the scope of this review. There is still a lack of consensus regarding surgical approaches to XAWs other than limiting debridement. CT scans can be helpful to evaluate abscesses or sinus tracts that may require debridement. After limited debridement, BTM can be placed over XAWs in the operating room with quickly dissolvable sutures. BTM is made of biodegradable synthetic material and is stable for approximately 18 months, allowing optimization of addiction care and social determinants of health for PWUD. Based on experiences from a local burn surgery team, BTM offers a better risk profile against wound infection [18, 19], compared with another commonly used dermal substitute [80].

When PWUD can abstain from illicit substance use for 6 weeks, wound closure with skin grafting can be considered [19]. This approach shares principles with other proposed surgical approaches for XAWs [30, 32]. However, it stands out for its harm reduction focus with realistic temporizing strategies. In addition, physical and occupational therapy commonly used in burn injury rehabilitation to preserve function and minimize disability may be employed for PWUD with XAWs [81].

## LONGITUDINAL AND RECIPROCAL MULTIDISCIPLINARY CARE

Care for people with XAW spans across different specialties, locales, and times. Regular and ad hoc citywide discussions in Philadelphia aim to harmonize treatment approaches. We need to improve the reciprocal hand-offs between community care organizations and various medical facilities. This includes referral to the hospitals from the community and postdischarge care transition. Low-barrier services offering care coordination, flexible hours, and walk-in appointments mitigate many barriers to care for PWUD [82]. Currently, several Philadelphia low-barrier wound care programs offer services and supplies free of charge [83]. However, experiences in the community suggest that the capacity of these services and supplies still needs expansion.

There is an urgent need for integrated programs to provide wound care, addiction care, and ID evaluation. A small number of low-barrier wound care clinics are staffed by ID physicians, but many other wound care clinics do not prescribe medications. Additionally, many medical offices do not offer wound care [84]. Furthermore, wound care is best incorporated into services that PWUD are already utilizing. Prevention Point Philadelphia, the largest syringe services program in the area, has been offering wound care since 2015 [7]. Some opioid treatment programs (methadone clinics) also offer wound care. Wound care in substance detoxification and rehabilitation programs would be immensely beneficial because many PWUD are refused admission due to XAWs. Although not primarily focusing on wound care, an ID–addiction longitudinal care model may serve a blueprint for an integrated care program [69]. Enhanced reimbursement may be a strategy to facilitate the incorporation of wound care into the existing infrastructure. Unfortunately, there is no specific *International Classification of Diseases, Tenth Revision* diagnosis code for XAWs, and wound care is reimbursed as a lump sum without itemized reimbursement for supplies (dressings, ointments, and medications). The City of Philadelphia shared common codes for diagnosis and billing [18] and started a pilot intensive inpatient program to offer inpatient addiction care (including methadone), long-term intravenous antibiotics, and wound care [85].

We desperately need to integrate the aforementioned services with outpatient surgical care. Even if PWUD with XAWs can be stabilized and cared for in the community, their access to surgery is limited. A medical–surgical integration program is much needed.

Finally, many PWUD with XAWs experience housing insecurity, which adds challenges to wound care. Unfortunately, some housing programs do not enroll people with complex XAWs. Expansion of housing-first programs [86] and medical respites [87, 88] with multidisciplinary care for XAWs must now be strongly considered.

## LIMITATIONS

This review has several limitations. First, the presented recommendations relied on institutional and local practices. The high prevalence and weight percentage of xylazine in IMF samples in Philadelphia make it impossible to study pathogenesis and clinical presentation of XAWs with control subjects who have little xylazine exposure. Despite recommending BTM as a bridge intervention for XAWs, it was based on experiences from a local burn surgery unit [19, 24]. Except for limited debridement, there is a lack of consensus regarding surgical approaches to XAWs. Second, our recommendations may not be generalizable to settings with fewer resources related to wound care, peer navigation, or addiction medicine. Due to the complexity of multidisciplinary care, we are unable to provide individualized alternative recommendations. Third, the recommended antibiotic choices may not be applicable to other institutions with different antimicrobial resistance patterns.

## FUTURE DIRECTIONS

Research into XAWs is much needed. Here we propose areas for prioritization in future work:

1. The pathogenesis and natural history of XAWs, as well as the prevalence of and risk factors for XAWs.
2. Accessible drug checking, which may help provide feedback to substance suppliers and facilitate safer supplies [89–92].
3. Best medical and surgical approaches: the determination of candidacy and timing of surgical interventions; choice of surgical interventions to cover XAWs after BTM; different approaches to wounds on the extremities versus not; strategies to avoid amputation; antibiotic treatment course as part of longitudinal care; and the minimally required degree and duration of substance abstinence before surgical interventions.
4. Reducing healthcare utilization and facilitating longitudinal multidisciplinary care: telemedicine wound care consultation; a cross-institutional registry of live wound assessments and treatment plans for each person to avoid inconsistent approaches and unnecessary hospital admissions; and implementation models for outpatient multidisciplinary care before definitive surgical intervention.

## CONCLUSIONS

Xylazine-associated wounds may become increasingly encountered as xylazine becomes more prevalent in the substance supply. We recommend multidisciplinary and low-barrier care across locations and time; aggressive treatment of withdrawal and pain; empirical MRSA and GAS treatment for infected XAWs; limited sharp debridement; and contingency antibiotic plans. Studies are urgently needed to inform best practices.

## Supplementary Material

ofaf299_Supplementary_Data

## Supplementary Data

Supplementary materials are available at *Open Forum Infectious Diseases* online. Consisting of data provided by the authors to benefit the reader, the posted materials are not copyedited and are the sole responsibility of the authors, so questions or comments should be addressed to the corresponding author.

## Notes

***Acknowledgments.*** The authors thank Drs Ines Lin and Sara Schultz for their review of and comments on the manuscript.

***Potential conflicts of interest.*** All authors: No reported conflicts.
