# Supplementary material for: Multidisciplinary Guidance to Care for Persons With Xylazine-Associated Wounds
Source: Open Forum Infect Dis. 2025 May 15;12(6):ofaf299. doi: 10.1093/ofid/ofaf299 (PMC12125673; doi:10.1093/ofid/ofaf299)
Supplement: ofaf299_Supplementary_Data [file ofaf299_supplementary_data.docx]

**Supplementary Table 1. Sample clinical scenarios and recommended management.**

| **Clinical scenario** | **Systemic antibiotics** | **ID consultation** | **Wound care consultation** | **Surgery consultation** |  |
| --- | --- | --- | --- | --- | --- |
| - Afebrile patient with severe pain from the wound. - Eschar covering the wound; minimal discharge, swelling, fluctuance, or surrounding erythema. - White blood cell count <15,000. - Hemodynamically stable. | Likely not needed. | Likely not needed. | Likely not needed. Bedside RN for wound care. | Likely not needed. Consider debriding eschar to facilitate healing. |  |
| - Afebrile patient with variable degree of pain from the wound. - Purulence, drainage from the wound, erythema or fluctuance in/around the wound. - White blood cell counts <15,000 **AND** - Hemodynamically stable. | Oral antibiotics. | Likely not needed. | Yes. | Consider if there are sinus tracks or abscesses. |  |
| - Febrile patient with variable degree of pain from the wound. - Purulence, drainage from wound, erythema or fluctuance in/around the wound. - White blood cell count >=15,000 **OR** - Hemodynamically unstable. | | IV antibiotics. | Yes. | Yes. | Consider if there are sinus tracks or abscesses. |
| - A febrile patient with variable degree of pain from the wound. - Chronically exposed bone or tendon **WITHOUT** an imminent plan to cover the wound. | Antibiotic therapy if there is local skin/soft tissue infection. **Do not** commit to long-term antibiotics for osteomyelitis unless there is a plan for imminent debridement and closure. | Consider. | Yes. | Only when the wound is acutely worsened with infected tissue or abscess to be debrided or drained. |  |
| - Rapidly progressing infection, or concern for necrotizing infection. | | IV antibiotics. | Yes. | Yes. | Yes. |
| - The patient has large abscesses, osteomyelitis, septic arthritis, bacteremia, infective endocarditis, or epidural abscess. | IV/oral antibiotics per ID. | Yes. | Yes. | When source control is needed. |  |

ID: infectious diseases; RN: registered nurse; IV: intravenous.
